# Distinct Risk Factors Contribute to Variations in Early Life Eczema Prevalence in Ethnically Chinese Children From Shanghai and Singapore

**DOI:** 10.1111/cea.70109

**Published:** 2025-07-22

**Authors:** Hui Xing Lau, Qian Chen, Qai Ven Yap, Yun Huang, Noor Hidayatul Aini Suaini, Yiong Huak Chan, Le Duc Huy Ta, Gaik Chin Yap, Lynette Pei‐Chi Shek, Elizabeth Huiwen Tham, Bee Wah Lee, Hugo Van Bever, Anne Eng Neo Goh, Kok Hian Tan, Fabian Kok Peng Yap, Yap Seng Chong, Keith M. Godfrey, Johan Gunnar Eriksson, Ying Tian, Jun Zhang, Evelyn Xiu Ling Loo

**Affiliations:** ^1^ Institute for Human Development and Potential (IHDP), Agency for Science, Technology and Research (A*STAR), 30 Medical Drive Singapore Republic of Singapore; ^2^ Ministry of Education‐Shanghai Key Laboratory of Children's Environmental Health Xinhua Hospital, Shanghai Jiao Tong University School of Medicine Shanghai China; ^3^ Department of Biostatistics, Yong Loo Lin School of Medicine National University of Singapore Singapore Singapore; ^4^ Department of Paediatrics, Yong Loo Lin School of Medicine National University of Singapore Singapore Singapore; ^5^ Human Potential Translational Research Programme, Yong Loo Lin School of Medicine National University of Singapore Singapore Singapore; ^6^ Khoo Teck Puat‐National University Children's Medical Institute National University Hospital, National University Health System Singapore Singapore; ^7^ Department of Paediatrics KK Women's and Children's Hospital (KKH) Singapore Singapore; ^8^ Department of Maternal Fetal Medicine KK Women's and Children's Hospital (KKH) Singapore Singapore; ^9^ Duke‐NUS Medical School Singapore Singapore; ^10^ Lee Kong Chian School of Medicine Nanyang Technological University Singapore Singapore; ^11^ Department of Obstetrics & Gynaecology, Yong Loo Lin School of Medicine National University of Singapore and National University Health System Singapore Singapore; ^12^ School of Human Development and Health, Faculty of Medicine University of Southampton Southampton UK; ^13^ NIHR Southampton Biomedical Research Centre University Hospital Southampton NHS Foundation Trust and University of Southampton Southampton UK; ^14^ MRC Lifecourse Epidemiology Unit, Faculty of Medicine University of Southampton Southampton UK; ^15^ Folkhälsan Research Center Helsinki Finland; ^16^ Department of General Practice and Primary Health Care University of Helsinki Helsinki Finland; ^17^ International Peace Maternity and Child Health Hospital Shanghai China

**Keywords:** allergy, eczema, paediatric study, Shanghai, Singapore

**Abbreviations**
ARR: adjusted risk ratio
CI: confidence interval
GUSTO: Growing Up in Singapore Towards healthy Outcomes
IQR: interquartile range
SBC: Shanghai Birth Cohort
SD: standard deviation

**Summary**

- We compared the epidemiology of eczema in ethnically Chinese children from China and Singapore.
- Maternal history of allergy in children born vaginally was a unique risk factor in Singapore.

To the Editor,

In recent decades, there has been a sharp rise in childhood eczema, especially in the first year of life. Genetic factors alone are insufficient to explain the rapid and non‐uniform changes in prevalence across regions. This points towards the involvement of environmental exposures and gene–environment interactions in eczema development. The early life environment is significant in shaping eczema development as genetically comparable populations exhibit variations in disease patterns [1].

There are emerging recommendations to improve the present generic approach to eczema management by taking into account ethnic differences in eczema aetiology [2]. We propose further enhancement to personalise eczema management and enhance efficacy by considering the local early life risk factors, even within ethnically similar populations. By targeting the earliest precursors of eczema, the aim is to halt the atopic march and reduce the burden of eczema and associated allergic comorbidities in children.

In this study, we compared the epidemiology of eczema in the first year of life in ethnically Chinese children from the Shanghai Birth Cohort (SBC) from China and the Growing Up in Singapore Towards Healthy Outcomes (GUSTO) cohort from Singapore. Demographic and lifestyle information beginning from pregnancy was collected using interviewer‐administered questionnaires in SBC and GUSTO, from which we identified 13 predictors with aligned definitions in both cohorts.

At 12 months, SBC parents were asked ‘Did your child ever had rashes since birth (including eczema and milk allergy)?’ and ‘What is the doctor's diagnosis of the rash (eczema/milk allergy/atopic dermatitis/others/not diagnosed)?’. Eczema was defined by positive responses to both rash occurrence and doctor's diagnosis of eczema or atopic dermatitis. The modified International Study of Asthma and Allergies in Childhood questionnaire was administered at 3, 6 and 12 months for GUSTO participants. Eczema was defined by a positive response to ‘Has your child ever been diagnosed with eczema?’. Ethics approval was obtained from the ethics committee of Shanghai Xinhua Hospital (XHEC‐C‐2013‐001, approved on 7 January 2013) for SBC and SingHealth Centralised Institutional Review Board (2009/280/D, approved on 2 March 2009) and the National Healthcare Group Domain Specific Review Board (D/2009/021, approved on 26 February 2009) for GUSTO. Written informed consent was obtained from all participants/the parents. All analyses were performed using SPSS 26.0 and STATA BE 17.

Of 3720 mother–child pairs from SBC and 691 ethnically Chinese mother–child pairs from GUSTO, 2341 (62.9%) and 520 (75.3%) from SBC and GUSTO, respectively, were included in this study after excluding participants without data on eczema outcomes by 12 months or non‐singleton pregnancies. The weighted prevalences of eczema by 12 months were 31.3% (95% CI 29.4–33.3) in SBC and 16.1% (95% CI 11.6–21.8) in GUSTO [3].

No significant associations were observed between all risk factors studied and eczema development in adjusted multivariate Poisson regression analysis in SBC (Table 1). In the GUSTO cohort, longer duration of gestation (ARR 0.75 [0.59–0.93]) was associated with a lower risk of eczema development, while a maternal history of allergy (ARR 3.4 [1.5–8.0]) was associated with a higher risk of eczema development. Moreover, in GUSTO, a maternal history of allergy was associated with a higher risk of eczema in children born vaginally (ARR 3.2 [1.6–6.4]) while no association was observed for children born by caesarean section (ARR 0.83 [0.30–2.32]) in the GUSTO cohort. This finding was not observed in SBC.

**TABLE 1:** Analysis of the associations between risk factors and eczema development in first year of life in SBC, Shanghai, China (recruited during pregnancy from 2013 to 2016) and GUSTO, Singapore (recruited during pregnancy from 2009 to 2010).

	SBC unadjusted		GUSTO unadjusted		SBC adjusted^a^			GUSTO adjusted^b^			*p* ^c^
	RR (95% CI)	*p*	RR (95% CI)	*p*	RR (95% CI)	*p*	Wald statistics	RR (95% CI)	*p*	Wald statistics	
Maternal age at recruitment	0.99 (0.97–1.01)	0.37	1.04 (0.99–1.09)	0.13	0.99 (0.96–1.01)	0.23	1.45	1.04 (0.98–1.11)	0.24	1.37	0.15
Pre‐pregnancy BMI	1.00 (0.97–1.02)	0.79	1.03 (0.97–1.10)	0.39	1.01 (0.99–1.04)	0.34	0.91	1.05 (0.97–1.13)	0.25	1.32	0.33
Nulliparous	1.0 (0.8–1.3)	0.69	1.5 (1.0–2.3)	0.05	0.99 (0.77–1.27)	0.91	0.01	1.4 (0.8–2.4)	0.26	1.28	0.18
Female	0.86 (0.74–1.01)	0.06	0.75 (0.49–1.15)	0.19	0.87 (0.74–1.03)	0.11	2.59	0.89 (0.52–1.52)	0.67	0.18	0.94
Less than university education	0.99 (0.85–1.15)	0.86	0.58 (0.38–0.89)	< 0.05	0.97 (0.81–1.16)	0.73	0.12	0.68 (0.38–1.22)	0.20	1.64	0.35
Ever or current smoker	1.3 (0.9–2.0)	0.15	1.0 (0.5–2.1)	0.98	1.5 (1.0–2.4)	0.07	3.24	1.5 (0.5–4.0)	0.47	0.52	> 0.99
Environmental smoke during pregnancy	1.0 (0.9–1.2)	0.72	0.79 (0.47–1.33)	0.38	1.1 (0.9–1.3)	0.38	0.76	0.75 (0.37–1.52)	0.43	0.64	0.35
Alcohol consumption before/during pregnancy	0.94 (0.75–1.18)	0.62	1.2 (0.8–1.9)	0.33	0.90 (0.69–1.19)	0.47	0.52	1.0 (0.6–1.7)	> 0.99	0	0.74
Caesarean delivery	1.0 (0.9–1.2)	0.77	1.0 (0.7–1.7)	0.85	0.93 (0.74–1.18)	0.56	0.34	1.7 (0.7–4.0)	0.22	1.5	0.09
GA	1.01 (0.96–1.06)	0.80	0.89 (0.79–1.00)	0.05	0.99 (0.93–1.06)	0.82	0.05	0.75 (0.59–0.93)	< 0.05	6.49	< 0.05
Birthweight	1.1 (0.9–1.3)	0.37	1.0 (0.6–1.7)	0.88	1.0 (0.9–1.1)	0.66	0.20	2.1 (1.0–4.5)	0.05	3.72	< 0.05
Use of antibiotics by M12	1.1 (0.9–1.3)	0.19	1.3 (0.8–2.1)	0.24	1.0 (0.9–1.3)	0.69	0.16	1.7 (0.8–4.0)	0.20	1.65	0.10
Maternal history of allergy X mode of delivery								0.28 (0.09–0.89)	< 0.05		
Maternal history of allergy	1.1 (0.9–1.3)	0.26	2.0 (1.3–3.1)	< 0.05	1.0 (0.8–1.3)	0.77	0.08	3.4 (1.5–8.0)	< 0.05	8.22	< 0.001
Subgroup: caesarean	1.3 (1.0–1.5)	< 0.05	0.86 (0.39–1.90)	0.71	1.2 (0.9–1.5)	0.13	2.24	0.83 (0.30–2.32)	0.73	0.12	
Subgroup: vaginal	0.97 (0.79–1.20)	0.79	3.0 (1.7–5.1)	< 0.001	1.1 (0.8–1.3)	0.68	0.18	3.2 (1.6–6.4)	< 0.001	11.01	

*Note:* Two‐sided *p* values are presented. Interaction between maternal history of allergy and mode of delivery were included in the model but not significant for SBC. The interaction term was derived from the multiplication of maternal history of allergy (1 = yes, 0 = no) by mode of delivery (1 = caesarean section, 0 = vaginal delivery). Interaction for maternal history of allergy and mode of delivery: *p* = 0.496 for SBC. Interaction between maternal history of allergy and use of antibiotics were included in the model but not significant. Interaction for maternal history of allergy and use of antibiotics: *p* = 0.798 for SBC and *p* = 0.593 for GUSTO.

Abbreviations: BMI, Body‐mass index; GA, Gestational age; GUSTO, Growing Up in Singapore Towards healthy Outcomes cohort; M12, age 12 months; SBC, Shanghai Birth Cohort.

^a^ 1859 out of 2341 (79.4%) SBC subjects were included in the adjusted analysis.

^b^ 377 out of 520 (72.5%) GUSTO subjects were included in the adjusted analysis.

^c^ *p* value for the difference between the two cohorts in the adjusted model.

To our knowledge, this is the first study to examine eczema prevalence and common risk factors among genetically comparable ethnic Chinese children residing in distinct geographical locations. Our findings highlight a potential unique risk factor in Singapore, namely maternal history of allergy in children born vaginally. While the role of maternal history of allergy is strongly recognised, current research has mostly established it as a non‐modifiable risk factor linked to the inheritance of high‐risk gene variants. There is no consensus on the involvement of other mechanisms, including cellular immunity, immunoglobulin E, inflammatory cytokines and microbiome [4, 5]. In support of our findings, a study in Jiangsu, China, reported a stronger association between maternal history of allergy and eczema in children born via vaginal birth as compared to caesarean section [6]. We hypothesise that this mechanistic pathway was more prominent in GUSTO as substantially higher proportions of mothers delivered vaginally than in SBC.

In addition, a longer gestational age may also exert stronger protection against childhood eczema in Singapore versus Shanghai, perhaps due to climatic differences, with a more robust skin epithelium at birth protecting against environmental insults in heat‐stressed Singapore. A higher gestational age may promote infant skin maturation and induce differential expression of skin protein biomarkers in infants. Notably, filaggrin‐processing proteins were found to be downregulated in preterm as compared to full‐term neonates [7].

This is a unique study featuring comprehensive profiling of participants from two mother‐offspring cohorts, allowing a range of demographic and risk factors to be aligned and compared. However, the definition of eczema was not identical. Another limitation is the use of parental reports of symptoms to assess eczema in children. However, we minimised recall bias and subjectivity through interviewer‐administered structured questionnaires. In addition, we were unable to identify risk factors in SBC despite the higher prevalence of eczema. This may be explained by the limited panel of common study variables from SBC and GUSTO studied, as well as the different initiation years of the cohorts. Our findings support more in‐depth investigations of the possible imprinting of allergic inflammation during vaginal birth in high‐risk children.

## Author Contributions

**Hui Xing Lau:** writing – original draft (equal), writing – review and editing (equal), project administration (equal). **Qian Chen:** conceptualization (equal), methodology (equal), project administration (equal), writing – review and editing (equal). **Qai Ven Yap:** formal analysis (equal), writing – original draft (equal), writing – review and editing (equal). **Yun Huang:** methodology (equal), project administration (equal), writing – review and editing (equal). **Noor Hidayatul Aini Suaini:** formal analysis (equal), writing – original draft (equal), writing – review and editing (equal). **Yiong Huak Chan:** formal analysis (equal), writing – review and editing (equal). **Le Duc Huy Ta:** methodology (equal), project administration (equal), writing – review and editing (equal). **Gaik Chin Yap:** methodology (equal), project administration (equal), writing – review and editing (equal). **Lynette Pei‐Chi Shek:** methodology (equal), project administration (equal), writing – review and editing (equal). **Elizabeth Huiwen Tham:** methodology (equal), project administration (equal), writing – review and editing (equal). **Bee Wah Lee:** methodology (equal), project administration (equal), writing – review and editing (equal). **Hugo Van Bever:** methodology (equal), project administration (equal), writing – review and editing (equal). **Anne Eng Neo Goh:** methodology (equal), project administration (equal), writing – review and editing (equal). **Kok Hian Tan:** methodology (equal), project administration (equal), writing – review and editing (equal). **Fabian Kok Peng Yap:** methodology (equal), project administration (equal), writing – review and editing (equal). **Yap Seng Chong:** methodology (equal), project administration (equal), writing – review and editing (equal), funding acquisition (lead). **Keith M. Godfrey:** methodology (equal), project administration (equal), writing – review and editing (equal). **Johan Gunnar Eriksson:** methodology (equal), project administration (equal), writing – review and editing (equal). **Ying Tian:** methodology (equal), project administration (equal), writing – review and editing (equal). **Jun Zhang:** conceptualization (lead), methodology (lead), formal analysis (lead), project administration (lead), supervision (lead), writing – original draft (lead), writing – review and editing (equal). **Evelyn Xiu Ling Loo:** conceptualization (lead), methodology (lead), formal analysis (lead), project administration (lead), supervision (lead), writing – original draft (lead), writing – review and editing (equal).

## Conflicts of Interest

Y.S.C. has received reimbursement for speaking at conferences sponsored by Abbott Nutrition, Nestle and Danone. Y.S.C. and K.M.G. are part of an academic consortium that has received research funding from Bayer, Nestle and Danone. The other authors declare no conflicts of interest.

## Data Availability

Data will be made available on reasonable request from the corresponding author due to ethical and privacy limitations. Supporting Information on predictors not analysed in this study is available at https://doi.org/10.5281/zenodo.15718144.
